# Youth mental health and religiously framed radicalization in the Middle East and North Africa region: a review of evidence and gaps

**DOI:** 10.3389/fpsyt.2026.1782072

**Published:** 2026-03-25

**Authors:** Gabriel Andrade, Abderrahim Benlahcene, Dalia Bedewy

**Affiliations:** 1 Department of Basic Sciences, College of Medicine, Ajman University, Ajman, United Arab Emirates; 2 Department of Psychology, College of Humanities and Sciences, Ajman University, Ajman, United Arab Emirates; 3 Humanities and Social Sciences Research Center (HSSRC), Ajman University, Ajman, United Arab Emirates; 4 Department of Educational Psychology, Faculty of Education, Tanta University, Tanta, Egypt

**Keywords:** Middle East and North Africa, public mental health, radicalization, religiosity, youth mental health

## Abstract

This narrative mini-review examines how youth mental health intersects with religiously framed radicalization in the Middle East and North Africa (MENA), conceptualizing radicalization as a potential maladaptive response to unmet psychological needs in highly religious, rapidly changing societies. It addresses three questions: how youth mental health relates to religious radicalization, what research gaps concern religious fanaticism as a response to lack of proper mental health care, and which conceptual, clinical, and policy developments are most needed. Available evidence indicates that there is no single “mental illness–terrorist” profile; a minority of radicalized individuals present diagnosable disorders, while symptoms such as depression, trauma, substance misuse, and personality vulnerabilities can heighten susceptibility to rigid, exclusionary religious narratives when combined with psychosocial adversity and exposure to extremist content, whereas everyday religious involvement often provides meaning, social support, and prosocial norms that protect against violence. The mini-review highlights the predominance of Western or diaspora samples, the scarcity of longitudinal and community-based research in MENA, and the neglect of non-violent but psychologically harmful forms of fanaticism. It calls for integrating youth mental health into radicalization prevention as a public health priority, expanding biopsychosocial−spiritual care models and collaboration with religious authorities, and developing region-specific guidance for managing radicalization risk in routine, non-securitized clinical practice.

## Introduction

Radicalization of youth has become a salient mental health concern in the Middle East and North Africa (MENA), where large cohorts of adolescents and young adults are navigating economic uncertainty, rapid social change, and complex regional dynamics (1). Religiously framed radicalization is not only a security issue but also closely intertwined with psychological distress, identity formation, and the availability—or absence—of appropriate mental health care, with vulnerability to rigid, absolutist narratives often reflecting unmet needs for meaning, belonging, and containment of psychic pain during sensitive developmental periods. This makes the MENA region a crucial setting for examining how structural deficits in mental health systems may contribute to trajectories of religious fanaticism among youth (2), including those who never engage in violence but nonetheless experience significant intrapsychic and interpersonal suffering.

In this mini-review, religion refers to socially shared systems of beliefs, practices, and institutions organized around the sacred and embedded in particular traditions, while religiosity denotes the degree and style of individuals’ religious involvement, commitment, and experience, which can range from flexible and questioning to rigid and dogmatic (3, 4). We use religious framing to describe situations in which personal or political grievances, identity threats, or mental health difficulties are interpreted primarily through religious narratives, symbols, and authorities, such that religion becomes the main lens for making sense of suffering and prescribing solutions. In line with much of the violent-extremism literature, radicalization is treated as a process through which some youth come to adopt increasingly exclusionary or absolutist beliefs, whereas extremism designates the resultant ideological position characterized by rejection of pluralism and endorsement of sharp in-group/out-group boundaries, and terrorism is reserved for the deliberate use or threat of violence against civilians in order to achieve political, ideological, or religious goals (5–8). Throughout, we focus on religiously framed radicalization that may or may not culminate in violent extremism or terrorism, thereby distinguishing between non-violent yet psychologically harmful fanaticism and organized campaigns of political violence (9, 10).

Likewise, radicalization linked to socio-political disenfranchisement in the post-Arab Spring landscape—such as reactions to authoritarian retrenchment, unemployment, or sectarian polarization—is analytically distinguished from trajectories in which religiously framed radicalization functions primarily as a maladaptive psychological response to distress, shame, or unmet attachment needs that are processed through rigid, punitive interpretations of faith.

The Arab Spring protests that began in late 2010 and spread across several MENA countries were a pivotal regional moment that reshaped social discourse, public space, and youth engagement (11). Alongside various social and institutional changes, these upheavals created new opportunities for competing ideologies and actors—including religious movements with more radical orientations—to gain visibility and influence, both on the ground and online. In some settings, periods of instability and realignment were accompanied by heightened exposure to sectarian narratives, polarizing media, and transnational networks that framed local grievances in religious terms. For a subset of vulnerable young people, these conditions appear to have facilitated pathways into more rigid and exclusionary religious worldviews, especially where experiences of loss, dislocation, or untreated psychological distress were already present (12). The post–Arab Spring landscape can therefore be understood as one in which new venues for radicalization have opened (13), reinforcing concerns among clinicians, policymakers, and communities about the implications for youth mental health and social cohesion.

In this sense, the Arab Spring is treated in this minireview not as a discrete “cause” of religiosity-based extremism, but as a macro-political turning point that reconfigured opportunity structures for a wide range of radical trajectories, only some of which are primarily religiously framed. Many youth mobilizations and post-2010 radical currents in MENA have been driven by grievances about corruption, authoritarian retrenchment, unemployment, and police brutality, in which religious language functions mainly as a moral vocabulary for socio-political disenfranchisement rather than as the central object of belief change or psychopathology (14).

By contrast, the focus of this minireview is narrower: it is concerned with those pathways in which religious narratives, symbols, and authorities become the main medium through which psychological distress, identity threat, or unmet attachment needs are processed, giving rise to rigid, punitive, or self-sacrificial forms of faith that may or may not intersect with explicitly political projects. Conceptually, the phenomenon under study is therefore “religiously framed radicalization” of youth—defined as the adoption of exclusionary, absolutist religious worldviews that organize meaning, self-worth, and in-group/out-group boundaries—within a post-Arab Spring landscape where socio-political grievances are ubiquitous but do not, by themselves, suffice to explain why some distressed young people move into fanatic milieus while many others do not.

The MENA region is highly heterogeneous in terms of religiosity, exposure to conflict, and adherence to extremist interpretations of faith, and many societies are characterized by strong traditions of moderation, pluralism, and religiously grounded care for others (15). In numerous settings, everyday religious practice provides social support, prosocial norms, and meaning-making that buffer against despair and violence, yet for some youth facing adversity, social exclusion, or unaddressed mental health problems, there is a pull toward more rigid religious currents that offer certainty and a sense of mission, illustrating the dual role of religion as both a source of resilience and a potential vector for fanaticism that complicates psychiatric research and practice in the region.

Across many MENA countries, substantial gaps in mental health service provision—shortages of trained professionals, under-resourced community services, and persisting stigma around psychiatric help-seeking—complicate efforts to address youth distress linked to radicalization (16). Individuals often first turn to religious leaders, family elders, or traditional healers, and while governments increasingly recognize that youth radicalization has major public health and psychosocial consequences (17) and are exploring preventive approaches that integrate mental health promotion, early identification, and collaboration with religious institutions, systematic evaluation of such initiatives remains limited.

Against this backdrop, the present mini-review focuses on religiously framed radicalization of youth in the MENA region as a potential maladaptive response to unmet mental health needs within highly religious and rapidly evolving social environments. It aims to synthesize conceptual and empirical work at the intersection of psychiatry, radicalization studies, and the sociology of religion, while remaining sensitive to regional heterogeneity and avoiding reductionist explanations that either pathologize religiosity or ignore mental health dimensions. Specifically, this review addresses three guiding questions: (1) What are the main schools of thought and current controversies regarding the relationship between youth mental health and religious radicalization in MENA? (2) What key research gaps limit understanding of religious fanaticism as a response to lack of proper mental health care? and (3) What future conceptual, clinical, and policy developments might support more effective and culturally attuned prevention and intervention strategies in this field?

## Methods

A narrative mini-review is a form of knowledge synthesis that draws selectively but transparently on diverse literatures to answer a focused question, emphasizing conceptual integration and critical interpretation rather than exhaustive, protocol-driven coverage or quantitative meta-analysis (18, 19) In this article, we adopt a narrative, problem-focused mini-review design: we use targeted database searches and iterative citation chaining to identify influential empirical, clinical, and conceptual work on youth mental health, religiosity, and radicalization in MENA, then organize these materials into thematic clusters to map what is known, where findings converge or diverge, and which gaps remain most salient for practice and policy. Narrative mini-reviews of this kind are particularly suitable when the evidence base is heterogeneous in methods and outcomes, when key constructs (e.g., “radicalization” or “fanaticism”) are contested, and when the primary goal is to refine conceptual frameworks and generate clinically relevant hypotheses rather than to produce pooled effect sizes (20). Our approach is similar in spirit of Moghaddam & Sardoč’s, (21) narrative review on radicalization and mental health, which synthesizes epidemiological, clinical, and socio-political studies to clarify how different mechanisms linking mental health and extremism have been theorized and studied without attempting a formal systematic review or meta-analysis.

This mini-review followed a targeted search strategy to identify conceptual and empirical work at the intersection of youth mental health, religiosity, and radicalization in MENA. Between January and March 2025, we searched PubMed, PsycINFO, Scopus, and Web of Science, complemented by regionally focused databases (Al Manhal and Arab World Research Source) and forward–backward citation chaining from key reviews on mental health and violent extremism, religion and mental health, and radicalization research. Search strings combined terms related to radicalization and extremism (“radicalization,” “violent extremism,” “religious fanaticism,” “terrorism,” “online extremism”) with mental health and psychosocial terms (“depression,” “psychosis,” “post-traumatic stress,” “personality disorder,” “identity,” “help-seeking”) and regional identifiers (“Middle East,” “North Africa,” “MENA,” plus individual country names), without language restrictions but with a practical focus on English- and Arabic-language publications.

Across all sources, the initial search and citation screening yielded approximately 150 records once duplicates were removed, including empirical studies, reviews, conceptual papers, and major organizational reports. Titles and abstracts were screened for relevance to three core domains—(a) radicalization, violent extremism, or rigid religious fanaticism; (b) explicit mental health, psychosocial, or service-use content; and (c) a primary focus on MENA populations or Muslim-majority diaspora samples judged conceptually transferable to MENA youth. We excluded opinion pieces and editorials without empirical or clearly articulated conceptual contribution, purely securitized risk-profiling tools with no mental health or religiosity dimension, single-case legal or forensic reports without clinical data, and studies focused exclusively on non-religious forms of extremism or on adult samples where youth were not analytically distinguishable.

Within this corpus, the final set of 56 sources included a mix of clinical and epidemiological studies, systematic reviews and meta-analyses, policy and public health reports, and socio-historical and conceptual analyses relevant to MENA youth, religiosity, and radicalization. For analytic purposes, these materials were grouped into broad clusters (empirical mental health–radicalization studies, work on religion and mental health in Muslim-majority contexts, public mental health and service-gap analyses, and scholarship on the Arab Spring and institutional responses to extremism), and then read iteratively to identify convergent and divergent themes concerning pathways to religiously framed radicalization, patterns of help-seeking, and implications for youth mental health services in the region.

**Figure 1:**
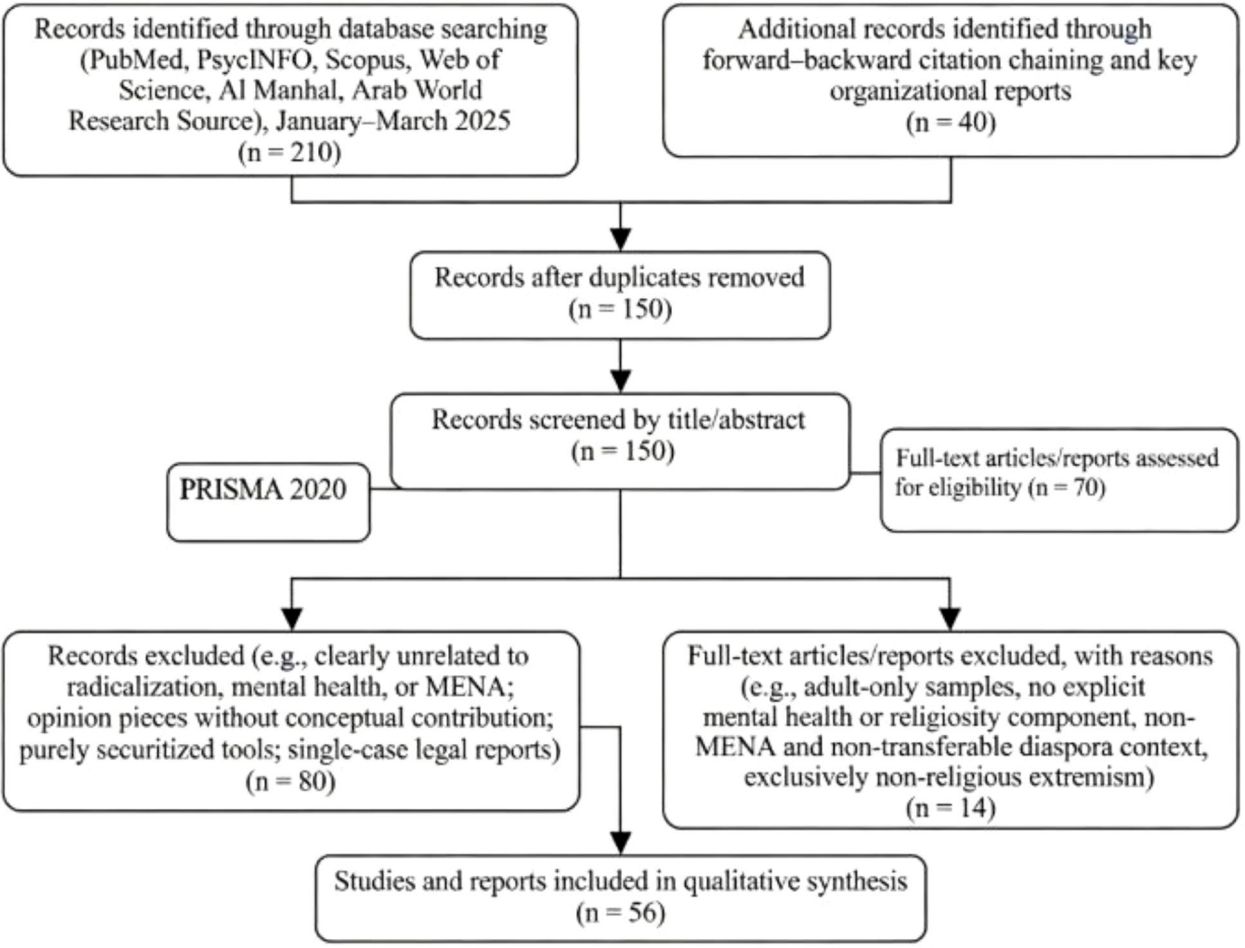
PRISMA diagram.

The PRISMA diagram guiding this procedure is presented in **Figure 1**.

## Results

The search and screening process yielded 56 publications that met the inclusion criteria for this mini-review. These comprised empirical studies, systematic reviews and meta-analyses, conceptual and socio-historical papers, as well as policy and public health reports relevant to youth, religiosity, and radicalization in MENA or closely comparable Muslim-majority contexts. For analytic purposes, the material was grouped into four broad clusters: (1) empirical mental health–radicalization studies, (2) work on religion and mental health in Muslim-majority settings, (3) public mental health and service-gap analyses, and (4) scholarship on the Arab Spring and institutional responses to extremism.

20 articles of the corpus consisted of empirical mental health–radicalization studies. These included cross-sectional surveys of psychiatric symptoms and extremist attitudes, clinical or forensic case series, qualitative interview studies with radicalized individuals or those at risk, and a small number of longitudinal or mixed-methods designs. Quantitative work most often relied on self-report symptom scales and risk factors (e.g., depression, trauma exposure, anger, perceived humiliation), while qualitative studies explored pathways into radical milieus, help-seeking trajectories, and the subjective meanings attached to religiously framed violence or non-violent fanaticism.

A second cluster (10 articles) comprised studies on religion and mental health in Muslim-majority contexts, including MENA countries and conceptually comparable diaspora samples. Most of these were observational (cross-sectional surveys, clinical samples, or community studies), examining associations between religiosity and mental health outcomes, patterns of reliance on imams or traditional healers, and the impact of religious stigma on help-seeking. Several also included qualitative or mixed-methods components that detailed how everyday religious practices function as sources of meaning, social support, and coping, while also documenting instances where rigid or punitive interpretations of faith contributed to shame, delayed treatment, or family conflict.

The third cluster (11 articles) included public mental health and service-gap analyses that addressed youth mental health systems, stigma, and patterns of informal vs. formal care in MENA. These were mainly narrative or scoping reviews, health systems reports, and policy analyses, sometimes supplemented by epidemiological data on prevalence and service use. Collectively, they highlighted shortages of trained professionals, under-resourced community services, limited integration of mental health into primary care, and high reliance on religious and traditional healing, thereby providing the structural backdrop for trajectories in which religiously framed radicalization can emerge as a maladaptive response to unmet needs.

Finally, the fourth cluster (15) consisted of socio-historical and political analyses of the Arab Spring, evolving radical currents, and institutional responses to extremism in the region. These were predominantly narrative or conceptual papers and book chapters, with some relying on secondary quantitative data and policy documents. They contextualized youth radicalization within broader dynamics of corruption, authoritarian retrenchment, unemployment, sectarian polarization, and shifting opportunity structures, and described how religious language can function both as a moral vocabulary for socio-political grievances and as a vehicle for more rigid, exclusionary worldviews that intersect with mental health vulnerabilities.

A summary table of all 61 selected articles is presented in **Table 1**.

**Table 1:** Summary table of articles reviewed.

#	First author (year)	Country/region focus	Article type	Methods/approach	Main focus/relevance to review
	Cluster 1: Empirical mental health–radicalization studies (n=20)				
1	Abbas et al. (22)	Northwestern Europe	Quantitative empirical study	Cross-sectional survey of humiliation, perceived power loss, and radicalization vulnerability	Identifies psychosocial drivers of radicalization vulnerability, illustrating mechanisms relevant to youth mental health and extremism.
2	Barak et al. (23)	Israel (traumatic loss)	Mixed/qualitative empirical study	Qualitative/quantitative study of ideological meaning-making after traumatic loss	Conceptualizes radicalization as a meaning-making response to traumatic bereavement.
3	Bélanger et al. (24)	International	Theoretical review	Narrative review and integrative model	Synthesizes work on self-sacrifice for a cause, linking personality and extremism.
4	Catapano et al. (25)	International	Systematic review	Systematic review of psychotic disorders and radicalization	Assesses relationship between psychotic disorders and radicalization processes.
5	Chabrol et al. (26)	France (young women)	Quantitative empirical study	Cross-sectional survey of Dark Tetrad traits and radicalization	Identifies personality profiles linked to radical attitudes among young women.
6	De Rosa et al. (27)	International	Book chapter (narrative review)	Narrative synthesis of radicalization and mental health within social-determinants lens	Integrates clinical and public mental health perspectives on radicalization.
7	Gill et al. (28)	International	Systematic review/meta-analysis	Systematic review and pooled analysis of mental health problems in violent extremism	Estimates prevalence of mental health diagnoses among individuals involved in violent extremism.
8	Hogg (29)	International	Theoretical review	Conceptual analysis of uncertainty, identity, and extremism	Proposes uncertainty-identity processes as pathways from threat to extremism.
9	Koehler (30)	International (adolescents)	Book chapter (narrative review)	Narrative synthesis with trauma-psychological perspective	Discusses violent extremism, mental health, and substance abuse among adolescents.
10	Leistedt (31)	International	Short conceptual/clinical paper	Conceptual discussion of radicalization process	Outlines stages and clinical aspects of radicalization process.
11	McGarry (32)	International	Commentary	Brief conceptual commentary	Argues terrorism is primarily political and cautions against over-pathologizing extremists.
12	Misiak et al. (33)	International	Systematic review	Systematic review of mental health, radicalization, and mass violence	Synthesizes evidence on links between psychiatric disorders, radicalization, and mass violence.
13	Mohammed et al. (34)	ISIS-affected regions	Quantitative empirical study	Cross-sectional study of multiple trauma, mental health, and reintegration expectations in IS-recruited youth	Explores how multiple traumatization and mental health problems shape reintegration prospects of putative juvenile terrorists.
14	Mohammed et al. (35)	Former ISIS fighters	Quantitative empirical study	Cross-sectional assessment of war trauma, mental health, aggression, and violent extremism	Analyses associations between trauma exposure, psychopathology, aggression, and extremist behavior.
15	Prats et al. (36)	Europe (lone-actor terrorism)	Forensic clinical paper	Forensic psychiatric assessment of lone-actor terrorist cases	Considers religious radicalization and lone-actor terrorism as matters for psychiatric evaluation.
16	Sarma (37)	International	Conceptual review	Narrative review of risk assessment and prevention	Discusses risk assessment for preventing progression from non-violence into terrorism.
17	Simi et al. (38)	USA (former White supremacists)	Qualitative life-history study	In-depth interviews with former extremists	Describes “identity residual” and the enduring effects of extremist involvement and mental health issues.
18	Trimbur et al. (39)	International	Systematic review	Systematic review of psychiatric disorders and terrorism/radicalization	Synthesizes prevalence of psychiatric disorders among radicalized individuals and terrorists.
19	Weatherston et al. (40)	International	Narrative review	Review of terrorism–mental illness relationship	Early review questioning simplistic associations between terrorism and mental illness.
20	Wolfowicz et al. (41)	International	Systematic review/meta-analysis	Field-wide systematic review and meta-analysis of risk/protective factors	Quantifies risk and protective factors for radicalization outcomes across studies.
	Cluster 2: Religion and mental health in Muslim-majority contexts (n=10)				
1	Abdulraof et al. (42)	Muslim clients (unspecified)	Empirical/clinical paper	Qualitative and practice-based discussion of Islamic-spirituality-integrated counselling	Describes culturally responsive Islamic spiritual interventions in mental health counselling for Muslim clients.
2	Alfain et al. (43)	Quranic perspective	Conceptual/theological article	Textual analysis of Quranic verses on patience and coping	Explores “patience” as a religious coping resource for mental problems.
3	Ali (44)	USA (imams)	Empirical survey	Cross-sectional survey of imams’ recognition and referral practices	Investigates how imams perceive, recognize, and refer mental illness in Muslim communities.
4	Ayoub et al. (45)	Lebanon	Observational clinical study	Clinical observational study of OCD with religious focus	Characterizes patients with religiously focused OCD and clinical implications.
5	Dein et al. (46)	UK/Middle East	Narrative review	Review of jinn possession within psychiatry	Discusses jinn-possession beliefs and implications for assessment and treatment.
6	Ghuloum et al. (47)	Eastern Mediterranean region	Narrative review	Regional review of religion and mental health	Summarizes evidence on religion, spirituality, and mental health in Eastern Mediterranean.
7	Huda et al. (48)	Muslim contexts	Mixed conceptual/empirical	Theoretical discussion with empirical illustration	Links tawakkul (trust in God) to self-regulation and religiosity, with implications for coping.
8	Koenig et al. (49)	West & Middle East	Narrative review	Comparative review of religion, spirituality, and mental health	Compares religious–spiritual influences on mental health in Western and Middle Eastern settings.
9	Rassool (50)	Islamic contexts	Monograph	Theoretical and clinical discussion	Explores evil eye, jinn possession, and mental health from an Islamic perspective.
10	Younis (51)	UK/Muslim communities	Conceptual/critical article	Critical political analysis	Critiques securitization and politicization of Muslim mental health in policy and practice.
	Cluster 3: Public mental health and service gap analyses (n=11)				
1	Alcalá et al. (17)	Global (incl. MENA)	Public health review	Narrative review with social determinants framework	Links social determinants of health to violent radicalization and terrorism from a public health perspective.
2	Bhui et al. (52)	UK/global	Conceptual/public health paper	Narrative review within public-health framework	Proposes a population-level public health approach to violent radicalization.
3	Kapidžić et al. (53)	Balkans & MENA	Policy/institutional analysis	Documentary and policy analysis of institutional approaches	Compares institutional responses to radicalization across Balkans, MENA, and beyond.
4	Mughal et al. (54)	International	Systematic review (empty review)	Systematic search for public mental health approaches to online radicalization	Reports absence of evaluated public mental health interventions targeting online radicalization.
5	Okasha et al. (16)	Arab world	Narrative health-systems review	Descriptive analysis of mental health services in Arab countries	Documents service gaps, workforce shortages, and cultural barriers to care in the Arab world.
6	Ozer et al. (55)	Globalization context	Book chapter	Conceptual analysis of globalization, disruption, and radicalization	Links intercultural contact, sociocultural disruption, and identity threat to radicalization.
7	Pollozhani et al. (2025)	Balkans & MENA	Empirical/policy paper	Comparative regional analysis of radicalization and violent extremism responses	Critiques securitized approaches and summarizes key regional findings on radicalization.
8	Remschmidt et al. (56)	Global children/adolescents	Narrative review	Global review of child and adolescent mental health care	Highlights worldwide inequalities and service gaps for youth mental health.
9	Rousseau et al. (2)	Youth in radicalization contexts	Brief review/commentary	Commentary on youth mental health amidst violent radicalization	Outlines current challenges in addressing youth mental health in radicalization settings.
10	Shafieioun et al. (57)	International	Conceptual article	Narrative, societal-level analysis	Frames radicalization as a societal phenomenon, emphasizing structural and cultural factors.
11	World Health Organization (58)	Global	Global report	Mixed-methods (surveys, administrative data, policy review)	Provides comprehensive global overview of mental health burden, systems, and reform, relevant to youth in MENA.
	Cluster 4: Arab Spring and institutional responses to extremism (n=15)				
1	Al-Badayneh et al. (15)	Arab youth	Empirical sociological study	Survey/quantitative analysis of social causes of Arab youth radicalizing	Examines social determinants and grievances linked to youth radicalization in Arab contexts.
2	Alegöz (59)	International	Literature review	Narrative review of radicalization research approaches	Summarizes key theoretical and methodological approaches in radicalization research.
3	Batzdorfer et al. (60)	International	Methodological review	Network-analytic review of radicalization research	Uses network methods to map themes and interconnections in radicalization literature.
4	Djamaluddin et al. (61)	Indonesia (Islamic university)	Empirical/qualitative	Qualitative/educational study of multicultural Islamic religious education	Explores deradicalization through multicultural Islamic education in university settings.
5	Doosje et al. (62)	International	Narrative review	Review of terrorism, radicalization, and de-radicalization models	Summarizes psychological models and empirical findings on radicalization trajectories.
6	Ducol (63)	International	Conceptual chapter	Conceptual argument for online/offline multidimensional approach	Advocates analyzing radicalization through combined online and offline social networks.
7	Ghannouchi (64)	North Africa	Book chapter	Conceptual/practice-oriented discussion	Discusses deradicalization through religious education programs.
8	Haas et al. (11), 11	Arab Spring region	Edited volume	Historical and political analyses of uprisings	Provides background on Arab Spring dynamics, youth mobilizations, and political transitions.
9	Isnawati et al. (65)	Indonesia (Santri)	Empirical/qualitative	Study of online deradicalization via digital literacy for Santri	Examines strengthening digital literacy as a tool for online deradicalization.
10	Lafi (14)	Arab world	Book chapter	Historical overview of Arab Spring as social movement	Situates Arab Spring within global history of social movements and political transitions.
11	Maiberg (66)	International	Narrative review	Review of methods for de-radicalization and disengagement	Summarizes practical tools and programs used to support de-radicalization.
12	Matesan (13)	MENA	Journal article	Qualitative/strategic analysis	Examines impact of Arab Spring on Islamist strategies and mobilization.
13	Neumann et al. (67)	International	Methodological review	Critical review of radicalization research quality	Evaluates methodological rigor and limitations of radicalization research.
14	Noueihed et al. (12)	MENA	Book	Journalistic and political analysis	Analyses revolution and counter-revolution in the Arab Spring and implications for youth.
15	Wibowo et al. (68)	International	Book chapter	Conceptual review of peace education	Discusses peace education as a strategy for deradicalization and conflict transformation.

## Discussion

### Schools of thought and controversies

The relationship between youth mental health and religiously framed radicalization in the MENA region has been shaped by several overlapping schools of thought, each supported by a growing but still methodologically limited empirical base. One influential line of research focuses on the “mental illness–terrorist” debate (40), examining whether psychiatric disorders are disproportionately represented among individuals involved in extremist milieus. A systematic review by Trimbur and colleagues, which screened 2,856 records and included 25 studies, reported that the prevalence of diagnosed psychiatric disorders among people at risk of radicalization, radicalized individuals, and terrorists ranges approximately from 3.4% to 48.5%, with particularly higher rates among lone-actor terrorists (39). The authors concluded that there is no consistent evidence for a simple, direct association between radicalization, terrorism, and psychiatric disorders, though subgroups with elevated prevalence clearly exist.

A complementary systematic review by Gill et al. pooled data from 19 samples (n = 1,705) and estimated that about 14.4% of individuals involved in violent extremism had a confirmed mental health diagnosis, with prevalence estimates across studies ranging from 0% to 57%, again highlighting heterogeneity rather than a single “terrorist psychopathology” (28). These syntheses have shifted the debate away from simplistic notions that all extremists are mentally ill, towards a more differentiated view in which psychiatric disorders may be one risk factor among many, variably expressed across contexts (32).

A second, partly overlapping, tradition emphasizes psychosocial and identity-based processes, viewing radicalization as embedded in trajectories of meaning-making and belonging, especially salient in adolescence and emerging adulthood (23). Systematic reviews linking mental health, radicalization, and mass violence suggest that factors such as prior trauma exposure (30), personality traits (e.g., narcissism, impulsivity) (26), and psychological problems (e.g., anger, perceived humiliation) (22) are present in a substantial proportion of cases, but typically in interaction with social and ideological influences rather than in isolation. For example, Simi and colleagues’ life-history interviews with former violent White supremacists (not MENA-based but influential for the field) found that a substantial percentage (around 40%) reported mental health problems before or during their involvement in extremism, often intertwined with substance use and histories of childhood adversity (38). Such findings have encouraged models in which youth radicalization is conceptualized as one possible, though far from inevitable, outcome of attempts to manage distress, shame, and identity uncertainty (29), especially when accessible mental health care and supportive social structures are lacking.

A third important axis of debate concerns the distinction between religiosity as a potential protective factor and religious fanaticism as a possible risk factor (41). Recent work on religion and mental health in the Eastern Mediterranean region underscores that, for many individuals, religious involvement is associated with hope, purpose, social support, and lower engagement in certain risk behaviors (47). At the same time, research also suggests that religious stigma surrounding mental illness—framing psychiatric symptoms as a punishment from God or a test of faith—can delay help-seeking and discourage open discussion of psychological difficulties (33). This dual role complicates clinical practice: ordinary religious devotion (especially in an Islamic context) may be beneficial for mental health, whereas more rigid, punitive, or exclusionary interpretations can contribute to distress and reduce engagement with professional care. Empirical data on specifically extremist or fanatic forms of religiosity in MENA youth remain sparse, but existing studies strongly suggest that the content and style of belief, and its embedding in social networks, matter more than religiosity per se (51).

Cultural psychiatry and public mental health perspectives add a further layer by situating both mental disorders and radicalization within broader social and service contexts (27). Some research suggests that high stigma and limited services in several countries lead many people to seek help first from religious figures, whose responses can either facilitate or obstruct contact with mental health professionals (57). This pattern is complemented by evidence from an “empty” systematic review of public mental health approaches to online radicalization, which found no evaluated interventions that explicitly integrate public mental health frameworks into responses to online extremist content, despite clear theoretical rationale and growing concern about youth exposure to such material (54). Together, these findings suggest that while mental health is widely acknowledged as relevant to radicalization processes, especially for youth, systematic, culturally sensitive public mental health strategies remain underdeveloped and under-evaluated.

Taken together, the empirical literature therefore supports a multidimensional framing in which psychiatric disorders, psychosocial adversities, and religious meaning-making interact in complex ways rather than following a single linear pathway (63). Research shows that clinically diagnosable mental disorders are neither necessary nor sufficient for radicalization, yet they may increase vulnerability in specific subgroups (25), particularly lone-actor offenders and individuals with histories of trauma or marginalization (36).

Available empirical work indicates that most evidence on psychiatric “subgroups” remains concentrated in Western and diaspora samples, where lone-actor terrorists and individuals with complex psychosocial needs show higher rates of diagnosed disorders than group-based militants. For example, Trimbur et al (39) systematic review found that psychiatric disorders among terrorists ranged from 3.4% to 48.5%, with elevated prevalence among lone actors, while still failing to demonstrate a simple mental illness–terrorism link overall. Similarly, Gill et al. (28) reported a pooled prevalence of confirmed mental health diagnoses of about 14.4% across 19 samples (n = 1,705) involved in violent extremism, again with heterogeneity and indications of higher rates in certain subgroups such as lone actors and those with prior system contact.

By contrast, there is very limited evidence characterizing analogous psychiatric subgroups among MENA youth specifically involved in radical milieus, and existing data are largely confined to post-conflict or carceral samples rather than community-based cohorts. A clinical study of adolescents and young adults incarcerated for ISIS-related offences in the Kurdistan Region of Iraq, for instance, documented extremely high rates of depression (89.8%) and PTSD (69.5%), suggesting a profile of heavy trauma burden and internalizing symptoms among this subset of “putative juvenile terrorists,” but did not allow fine-grained distinctions between lone actors and group-based offenders (34). Taken together, current evidence therefore permits cautious inference that trauma-exposed, justice-involved youth in MENA may constitute a particularly vulnerable subgroup, yet robust epidemiological data on psychiatric disorders among radicalized MENA youth outside custodial or former-combatant settings are still lacking, and most subgroup findings continue to derive from Western or diaspora populations rather than from young people living in the region itself.

Psychosocial and cultural-psychiatric perspectives, supported by regional data on religion, stigma, and help-seeking, indicate that in MENA contexts the availability and acceptability of mental health care, as well as the ways religious frameworks interpret suffering, are central to understanding how some youth move towards rigid, fanatic positions while others find in religion a source of resilience (53).

### Current research gaps

A striking feature of the existing literature is how little empirical work directly examines the intersection of youth mental health, religiosity, and radicalization within MENA settings. Systematic reviews of mental health and violent extremism draw overwhelmingly on samples from Europe, North America, and, to a lesser degree, South and Southeast Asia, with only a small subset of studies focusing on Arab-majority or broader MENA populations (55). Even in these cases, the samples are often drawn from migrants or diaspora communities living in Western countries rather than from young people residing in the region itself. As a result, key findings about the prevalence of psychiatric disorders among radicalized individuals, or about risk and protective factors, are frequently extrapolated from contexts with very different health systems, religious fields, and socio-political dynamics (37). There is a particular scarcity of longitudinal, community-based research following MENA youth over time to examine how mental health trajectories, exposure to adversity, patterns of service use, and religious engagement interact to shape susceptibility to extremist networks or, conversely, resilience against them. This gap limits the ability to distinguish transient ideological experimentation from more entrenched forms of fanaticism, and to understand how changes in mental state or access to care might alter radicalization pathways.

Closely related is the under-exploration of mental health service gaps and pathways to care in radicalization research. Public mental health and global mental health literatures consistently document substantial treatment gaps across many MENA countries, with WHO and regional reports indicating that a large majority of people with common mental disorders do not receive minimally adequate care, and that specialist services are often concentrated in urban centers (58).

Studies on religion and mental health in the Eastern Mediterranean further highlight the central role of religious leaders, traditional healers, and family networks as first-line responders to distress, and the persistence of stigma surrounding formal psychiatric services (49). Yet, these service-related variables are rarely measured in empirical work on radicalization, which tends to focus on ideological attitudes, criminal histories, or socio-demographic factors rather than on help-seeking trajectories or contact with mental health systems (67). There are virtually no studies that systematically map the sequence from early psychological distress through informal, often religiously framed care, to possible involvement in more rigid or extremist religious milieus among youth in the region. Without such data, it remains largely speculative to claim that deficits in mental health care contribute to radicalization, even though this hypothesis is intuitively compelling and frequently invoked in policy discussions.

In many MENA settings, informal help-seeking unfolds within dense family and neighborhood networks where spiritual idioms of distress are not abstract beliefs but part of everyday decision-making about suffering (50). When a young person begins to hear voices, withdraw socially, or show sudden behavioral changes, relatives may first ask whether someone has cast siḥr (magic), whether a jealous acquaintance has inflicted the 'ain (evil eye), or whether the person has been exposed to jinn in particular locations (such as cemeteries, abandoned buildings, or bathrooms). This interpretive process often involves elders, mothers, and sometimes female kin with reputations for “knowing” about such matters, who may recommend specific forms of ruqyah, visits to a local sheikh, or the burning of incense and use of Qur’anic verses in the household long before the possibility of a psychiatric consultation is raised (69). In close-knit communities, such attributions can be protective—offering a shared narrative that reduces blame on the individual—but they also make public recourse to biomedical care feel like an admission that the family has failed religiously or morally, which can be especially stigmatizing in contexts where marriage prospects and family honor are salient concerns (70).

These cultural idioms of distress also shape the temporal sequence and thresholds for help-seeking (46). Families may cycle through multiple spiritual and traditional remedies—repeated ruqyah sessions, changes in ritual practice, vows (nadhr), or seeking amulets (ḥijāb) from reputed healers—before considering a mental health professional, and psychiatrists are often consulted only after crises such as a suicide attempt, aggressive outburst, or involvement with authorities (71). For some youth, this means that early warning signs of depression, psychosis, or substance misuse are reframed as tests of faith or signs of spiritual attack, leading to exhortations to pray more, recite specific sūrahs, or increase modesty rather than invitations to speak openly about psychological pain. At the same time, these idioms can be integrated into collaborative care when clinicians acknowledge them respectfully and work with families and religious figures, rather than dismissing them as mere superstition, to negotiate pathways that allow simultaneous engagement with spiritual resources and formal mental health services (72).

These models of suffering are also relevant for understanding pathways into religiously framed radicalization, because they shape which authorities are trusted and which interventions are perceived as legitimate when youth experience intense psychological pain or behavioral change. In some cases, rigid preachers or informal religious actors may recast symptoms attributed to jinn or 'ain as signs of a broader moral and spiritual struggle, portraying surrounding social environments—such as mixed-gender schools, secular entertainment, or peers who are perceived as lax in their religious practice—as corrupting influences that endanger faith and moral purity, and urging withdrawal from these spaces in favor of more tightly controlled religious milieus (73). Such trajectories suggest that explanatory models centered on jinn and 'ain can, under certain conditions, contribute to treatment delays and amplify stigma, while also creating openings through which vulnerable young people are drawn into more closed, high-demand religious networks before they ever encounter formal mental health services.

Methodological and conceptual limitations further complicate interpretation of the existing evidence. Reviews repeatedly emphasize wide heterogeneity in how “radicalization,” “extremism,” and “terrorism” are defined and operationalized, ranging from attitudinal support for extremist ideas to actual participation in violent acts (59). In some studies, conservative or fundamentalist religious beliefs are implicitly treated as proxies for extremism, which risks conflating non-violent religiosity with support for violence and obscuring important distinctions between devotion, rigidity, and fanaticism (60). Many empirical investigations rely on small, highly selected samples—such as individuals in prison, under security services’ supervision, or referred to specialized forensic clinics—which may not be representative of the broader population of youth who encounter radical ideas or engage in non-violent forms of fanaticism. Cross-sectional designs dominate, limiting insights into temporal ordering and causal mechanisms (31), and few studies use culturally validated psychiatric instruments or nuanced measures of religiosity and spirituality tailored to MENA contexts. These limitations make it difficult to draw robust conclusions about how mental health problems, religious worldviews, and exposure to extremist narratives are related in everyday community settings.

Finally, there is a notable neglect of non-violent yet psychologically harmful forms of religious fanaticism in the empirical literature. Most studies center on violent extremism as defined by participation in or support for terrorist groups and attacks, leaving aside patterns of rigid, punitive, or self-sacrificial religiosity that may not lead to violence but can nonetheless be associated with self-harm, social isolation, family conflict, or intense internalized shame (24).

In clinical practice across several MENA countries, such non-violent fanaticism can present as severe religious scrupulosity in adolescents and young adults who experience intrusive doubts about the correctness or purity of their wudu (ablution), prayer, or Qur’an recitation, leading to highly time-consuming rituals, repeated washing until skin breakdown, and distress if every perceived error is not corrected (45). Rather than being framed as a potentially treatable form of obsessive–compulsive disorder, these youths and their families may interpret the symptoms as evidence of exceptional piety or of satanic whispers that must be countered through further ritual intensification, which can normalize impairment, delay referral, and reinforce rigid cognitions about sin, contamination, and divine punishment that intersect with, but do not necessarily culminate in, overt support for violence.

Other MENA youth show non-violent fanatic trajectories characterized by progressive social withdrawal, school dropout, or refusal to interact with peers and extended family deemed religiously inadequate because of music, dress, or perceived moral laxity, sometimes accompanied by extreme self-criticism, fasting, or self-denial driven by fears of falling into unbelief or major sin. These patterns may be praised in some environments as signs of heightened religious consciousness, yet they are often associated with depression, anxiety, family conflict, and a shrinking of the social world to a small circle of like-minded believers or online communities, illustrating how rigid purity and sin concerns can produce substantial psychological harm and relational rupture without crossing the threshold into violent extremism.

Work on religion and mental health in the Eastern Mediterranean points to the potential for religious stigma and fatalistic interpretations of distress to exacerbate symptoms and delay treatment (34), but this is rarely linked explicitly to questions of radicalization or extremist milieus. The narrow focus on violent outcomes risks missing important subthreshold phenomena in schools, universities, and online spaces, where youth may adopt extreme views about purity, sin, or out-groups that compromise their psychological well-being and relationships without ever attracting the attention of security services.

### Limitations

This narrative mini-review has some limitations that should be considered when interpreting its findings and recommendations. First, although the search strategy combined multiple databases and forward–backward citation chaining, it was not designed or registered as a full systematic review, and study selection involved informed but inevitably subjective judgments about relevance and conceptual transferability to MENA youth. The resulting corpus of 56 sources is therefore illustrative rather than exhaustive, and some pertinent work—particularly in local languages or grey literature—may have been missed.

Second, much of the empirical evidence on mental health and radicalization still comes from Western or diaspora samples, with relatively few longitudinal, community-based, or clinical studies focused specifically on youth in MENA. This limits the ability to draw firm causal inferences about how psychiatric disorders, psychosocial adversity, and religious meaning-making interact in the region, and it requires cautious extrapolation from contexts where service systems, political dynamics, and everyday religiosity may differ substantially.

Third, the available studies are heterogeneous in design, measures, and conceptualizations of both “radicalization” and “mental health,” which complicates comparison and synthesis. Many rely on cross-sectional surveys, self-report instruments, or small clinical and forensic samples, and very few examine non-violent but psychologically harmful forms of religious fanaticism or systematically track pathways from early distress through informal religious or traditional help-seeking into more rigid milieus. In addition, most public mental health and service-gap analyses provide broad system-level descriptions rather than evaluated interventions that integrate youth mental health and radicalization prevention.

### Potential future developments

Future work in this area will benefit from explicitly integrating mental health into broader radicalization prevention frameworks, particularly in relation to online environments. Public mental health approaches conceive online and offline radicalization as embedded in intersecting individual, family, community, and structural influences, rather than as isolated security problems (52).

Building on these insights, school and community-based programs in MENA could incorporate routine screening for depression, trauma symptoms, substance use, and suicidality, while also addressing religious meaning-making, identity development, and digital literacy through psychoeducation and group-based interventions (65). Embedding such initiatives within whole-of-society strategies for youth mental health, as advocated by recent WHO and UNICEF joint programs (68), would help position radicalization prevention within a broader agenda of promotion, prevention, and early intervention rather than narrow risk management.

Developing culturally and religiously sensitive care models will be essential for making these interventions acceptable and effective in MENA (61). Recent work on religion and mental health in the Eastern Mediterranean highlights the potential of the biopsychosocial−spiritual model to integrate faith-informed interventions with evidence-based psychiatric care, addressing misconceptions about the relationship between faith, illness, and autonomy (62). Such an approach is compatible with Islamic counseling frameworks and Islamic cognitive therapy (66), which adapt cognitive-behavioral principles to concepts like *tawakkul* (trust in God) (48) and *sabr* (patience) (43) within a structured therapeutic model (42). Empirical work with imams in Muslim communities shows that clergy can recognize severe mental illness and, when they have prior collaborative contact with clinicians, are more willing to support referrals and acknowledge the usefulness of psychiatric medication (44). Training religious leaders in basic mental health literacy, risk recognition, and referral pathways—while also equipping clinicians to engage respectfully with religious frameworks—could reduce stigma, counter extremist narratives that frame psychiatric care as religiously forbidden, and create more trusted entry points into care (64).

At the policy and system level, these directions imply increased investment in youth mental health services, integration of mental health into primary care, and explicit linkage of mental health promotion with violence prevention strategies in MENA. WHO and regional partners have already called for comprehensive child and adolescent mental health strategies and whole-of-society approaches that connect schools, communities, and digital platforms with health services, which could be adapted to include attention to radicalization risk (56). Region-wide collaborations involving WHO, professional associations, and academic networks could develop guidance on assessing and managing radicalization risk within ordinary clinical practice—emphasizing confidentiality, therapeutic alliance, and non-stigmatizing care, and avoiding the securitization of routine mental health encounters.

## Conclusion

This mini-review addressed three guiding questions and points to a multifactorial understanding of youth radicalization in the MENA region. Regarding how youth mental health relates to religious radicalization, the evidence indicates that there is no single “mental illness–terrorist” profile; rather, a minority of radicalized individuals present diagnosable disorders, while symptoms of depression, trauma, substance misuse, and personality vulnerabilities can heighten susceptibility to rigid, exclusionary religious narratives when combined with psychosocial adversity, exposure to extremist content, and specific belief styles, whereas everyday religious involvement more often functions as a source of meaning, social support, and prosocial norms that protect against violence.

Concerning the main research gaps on religious fanaticism as a response to lack of proper mental health care, current work remains heavily based on Western or diaspora samples, with very limited longitudinal and community-based research in MENA, little empirical mapping of pathways from early distress through informal religious or traditional help-seeking into potential fanatic milieus, minimal systematic inclusion of variables such as treatment gaps, stigma, and reliance on faith healers, and a marked neglect of non-violent but psychologically harmful forms of fanaticism expressed as self-harm, social withdrawal, family conflict, or internalized shame rather than overt violence.

Finally, with respect to future conceptual, clinical, and policy developments, the review underscores the need to integrate youth mental health into radicalization prevention as a public health priority, expand biopsychosocial−spiritual care models and structured collaboration with religious authorities, and implement mixed-methods, longitudinal, and participatory research alongside system-level reforms that increase investment in youth services, embed mental health in primary care, and develop region-wide guidance for managing radicalization risk within routine, non-securitized clinical practice.
